# Association of the HLA-DQA1*05 Allelic Gene Variants with Immunogenicity to Anti-TNF therapeutics—Important Differences Between Infliximab and Adalimumab

**DOI:** 10.1093/ecco-jcc/jjae172

**Published:** 2024-11-16

**Authors:** Nicola Ternette, Hanqing Liao, Jack Satsangi

**Affiliations:** School of Life Sciences, University of Dundee, Dundee, UK; Nuffield Department of Medicine, University of Oxford, Oxford, UK; School of Life Sciences, University of Dundee, Dundee, UK; Nuffield Department of Medicine, University of Oxford, Oxford, UK; Translational Gastroenterology and Liver Unit, Nuffield Department of Medicine, University of Oxford, Oxford, UK

Dear Editor,

We read the article published by Reppell et al.^1^ with interest. Here, the authors report the strongest association of the development of antibodies to adalimumab with HLA-DQA1*05:05 (and DRB1*01:02) in Crohn’s disease and ulcerative colitis patients. The authors further emphasize that HLA-DQA*05:01 is *not* associated with the development of antibodies to adalimumab.

These observations are entirely consistent with and represent an important replication of the findings of our reanalysis of the PANTS patient cohort, outlined in our 2020 publication in Gastroenterology, titled “Extended Analysis Identifies Drug-Specific Association of Two Distinct HLA Class II Haplotypes for Development of Immunogenicity to Adalimumab and Infliximab”.^2^ We demonstrated that individuals carrying the HLA-DQA1*05:05 are at risk to develop antibodies against both infliximab and adalimumab, while HLA-DQA1*05:01 is associated with the development of antibodies against infliximab only, and is *not* associated with the development of antibodies to adalimumab as had been previously incorrectly claimed.^3^

Our analysis underscored the critical value of employing 4-digit HLA resolution for correlation analyses, which Reppell et al. also advocate: We emphasize the need to avoid inappropriate and inaccurate over-simplification of the relationship between the HLA-DQA1*05 supertype allele and immunogenicity to anti-tumour necrosis factor (TNF) antibody therapeutics in inflammatory bowel disease. Variant-specific effects *must* be recognized to avoid misinterpretation of immunogenicity risks, ensuring accurate decision-making if these results are translated to clinical practice.

In addition, we also point out in our previous work that HLA genes are often not inherited independently, and that the resulting linkage disequilibrium leads to strong effects of co-inheritance: DQA1*05:05 is highly frequently co-inherited with DQB1*03:01 and DRB1*11:01, and therefore the latter alleles also significantly associate with the development of antibodies against adalimumab, as reported by both our own and the present study.

These considerations are critically important when considering the molecular mechanism underlying the HLA association with drug immune responses: HLA class II molecules are heterodimers that consist of two protein chains (alpha and beta), and the associated genes reported by gene association studies encode *only one* of the two chains of the functional HLA molecule. More advanced statistical modeling is needed to accurately determine which combinations of both alpha and beta chains pose the greatest risk to patients. Furthermore, neither the SERENE nor PANTS studies included information on the HLA-DP locus, and therefore the role and association of DP genes with anti-drug immune responses has yet to be determined.

We conclude that the molecular immunological processes that lead to the induction of antibodies in response to drug treatment must be interrogated further, in order to pave a way toward the design of improved biologics with reduced immunogenicity in the future.

## Data Availability

Links to the original data and manuscripts are referenced in the manuscript.

## References

[1] Reppell M , Zheng X, Dreher I, et al HLA-DQA1*05 associates with anti-TNF immunogenicity and low adalimumab trough concentrations in inflammatory bowel disease patients from the SERENE UC and CD studies. J Crohns Colitis 2024:jjae129. https://doi.org:10.1093/ecco-jcc/jjae129 PMC11725519 39162746

[2] Powell Doherty RD , Liao H, Satsangi JJ, Ternette N. Extended analysis identifies drug-specific association of two distinct HLA class II haplotypes for development of immunogenicity to adalimumab and infliximab. Gastroenterology 2020;159:784–7. 32275970 10.1053/j.gastro.2020.03.073

[3] Sazonovs A , Ahmad T, Anderson CA. Underpowered PANTS: a response to the conclusions of “Extended analysis identifies drug-specific association of two distinct HLA class II Haplotypes for development of immunogenicity to Adalimumab and Infliximab”. Gastroenterology 2021;160:470–1. 33022279 10.1053/j.gastro.2020.05.102

